# Mathematical Modelling of Endocrine Systems

**DOI:** 10.1016/j.tem.2019.01.008

**Published:** 2019-04

**Authors:** Eder Zavala, Kyle C.A. Wedgwood, Margaritis Voliotis, Joël Tabak, Francesca Spiga, Stafford L. Lightman, Krasimira Tsaneva-Atanasova

**Affiliations:** 1 Living Systems Institute, University of Exeter, Exeter EX4 4QD, UK; 2 EPSRC Centre for Predictive Modelling in Healthcare, University of Exeter, Exeter EX4 4QD, UK; 3 Centre for Biomedical Modelling and Analysis, University of Exeter, Exeter EX4 4QD, UK; 4 College of Engineering, Mathematics and Physical Sciences, University of Exeter, Exeter EX4 4QF, UK; 5 Institute of Biomedical and Clinical Science, College of Medicine and Health, University of Exeter, Exeter EX4 4PS, UK; 6 Henry Wellcome Laboratories for Integrative Neuroscience and Endocrinology, University of Bristol, Bristol BS1 3NY, UK

**Keywords:** hormone dynamics, regulatory networks, circadian rhythms, ultradian oscillations, chronotherapy, hybrid systems

## Abstract

Hormone rhythms are ubiquitous and essential to sustain normal physiological functions. Combined mathematical modelling and experimental approaches have shown that these rhythms result from regulatory processes occurring at multiple levels of organisation and require continuous dynamic equilibration, particularly in response to stimuli. We review how such an interdisciplinary approach has been successfully applied to unravel complex regulatory mechanisms in the metabolic, stress, and reproductive axes. We discuss how this strategy is likely to be instrumental for making progress in emerging areas such as chronobiology and network physiology. Ultimately, we envisage that the insight provided by mathematical models could lead to novel experimental tools able to continuously adapt parameters to gradual physiological changes and the design of clinical interventions to restore normal endocrine function.

## Highlights

Combining appropriate mathematical models with carefully designed experiments offers great potential to understand complex endocrine regulation at multiple levels of organisation.

Understanding the mechanisms underlying coordinated, rhythmic insulin secretion requires novel mathematical and computational methods that consider the pancreatic islet as a network of beta cells.

Mathematical models, in combination with experimental physiology, have uncovered the mechanisms by which glucocorticoid hormones exhibit normal ultradian pulsatility and respond rapidly to stressors, including during inflammation.

Supported by optogenetic experiments, a hypothalamic neural network comprising kisspeptin secretory neurons has been postulated as driving pulsatile GnRH dynamics involved in the regulation of the reproductive cycle.

The dynamic clamp, a hybrid system integrating electrophysiological measurements with mathematical modelling, enables the interactive manipulation of key parameters in real time.

## Understanding the Complexity of Endocrine Regulation Demands an Interdisciplinary Approach

Endocrine axes are the perfect example of complex physiological regulatory systems involving multiple levels of organisation (e.g., central nervous system, secretory glands, tissues, cells, hormones) and timescales [e.g., monthly rhythms, **circadian** (see Glossary) oscillations, **ultradian** fluctuations, fast responses]. These systems typically exhibit **nonlinear responses**, possess multiple components with several feedback loops, and are involved in crosstalk interactions with each other and other body systems (e.g., the immune and nervous systems, the digestive and reproductive apparatus). Endocrine axes are also highly dynamic, with hormone levels exhibiting complex temporal behaviour over short and long timescales that combines **sensitivity** with **robustness**, which allows adaptability to physiological challenges. More importantly, dysregulation of these dynamic processes (particularly when it is irreversible) can lead to disease.

Since the seminal work by Norbert Wiener in the mid-20th century, mathematical modelling has helped physiologists to understand how concepts such as negative feedback are key to homeostasis. In endocrinology, new mechanisms of dynamic active regulation have been uncovered to explain the ability to anticipate events and to quickly react to stimuli. Instead of stabilising set points within a certain range, endocrine axes generally control dynamic phenomena (e.g., hormone rhythms, neuron firing, body temperature). Notably, the efforts to uncover the regulatory mechanisms that sustain this ‘homeodynamics’, their robustness in the face of disturbances, their plasticity to adapt to new dynamic regimes (**allostasis**), and their disruption during disease have largely benefited from mathematics. Some of these benefits have been already described in several reviews. The review in [1] covers general principles of modelling in neuroendocrinology using the growth hormone system as an example, while a recent review by the same authors addresses the contributions of modelling to hypothalamic–pituitary neurosecretory systems [2]. The review in [3] describes in detail several mathematical modelling tools such as types of equations, analysis of their dynamic behaviour (e.g., **bistability**, oscillations), and approaches to deal with biological noise and systems with multiple timescales in view of applications in endocrinology. This demonstrates a growing interest in the use of quantitative tools and methods to investigate complex hormone dynamics, particularly in relation to stress, reproduction, and metabolism 4, 5, 6, 7, 8, 9. However, an increased appreciation of the insight that mathematical modelling can bring to experimental research could better inform the design of novel interdisciplinary approaches aimed at untangling the complexity of endocrine regulation.

In this review, we show how combining mathematical models with the appropriate experimental set up amounts to the best tool available to understand this complexity. Through examples from the metabolic, stress, and reproductive axes, we illustrate how models can provide insight on dynamic hormone regulation spanning several spatiotemporal scales and the key role that these quantitative models could play in the advancement of **chronomedicine**. Rather than present the vast and diverse array of mathematical models used in endocrinology, which we feel may be overwhelming, we choose instead to demonstrate how models have been used to answer specific questions. We also discuss an example of a new class of hybrid approaches: the dynamic clamp in electrophysiology, where real-time integration of mathematical modelling with experimental techniques can be used to understand the behaviour of secretory cells. Lastly, through a discussion of open research questions at the intersections between the metabolic, stress, and reproductive axes, we give a perspective of the field and how experimental and clinical research can benefit from mathematical modelling approaches.

## The Metabolic Axis: From Mechanisms of Secretion to Beta Cell Coordination and Beyond

**Figure 1:**
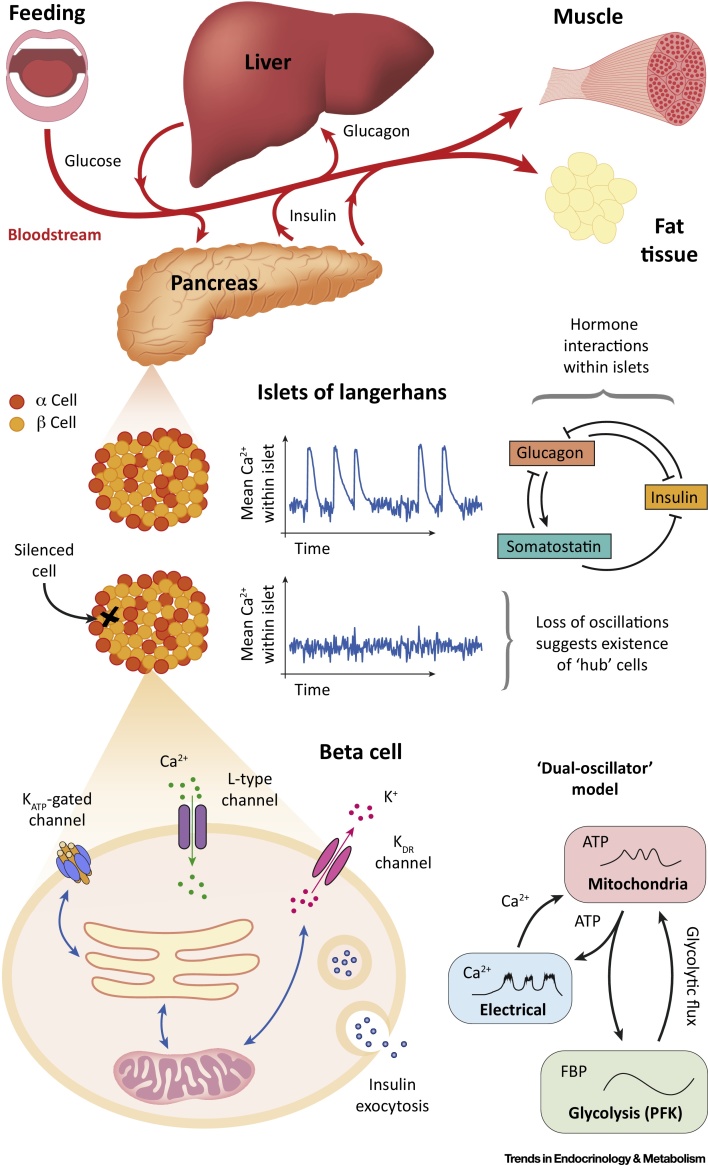
The Metabolic Axis. Regulation of blood plasma glucose levels is achieved primarily through the complementary actions of the hormones insulin, glucagon, and somatostatin. Insulin promotes the absorption of glucose from the blood by the liver and peripheral tissues, thus lowering the blood glucose concentration. In these tissues, glucose is then converted to glycogen or fat and subsequently stored. Glucagon plays the opposite role to insulin, encouraging tissues to transform these substrates back into glucose for secretion into the bloodstream. Somatostatin inhibits the secretion of insulin and glucagon by, respectively, beta and alpha cells, both of which reside in multicellular structures known as the islets of Langerhans, which are located in the pancreas. Mathematical models of beta cell behaviour typically account for the electrical activity originating from ion channels involved in insulin secretion. Recent models have also accounted for beta cell metabolism, including, for example, the glycolytic activity and mitochondrial components shown in the ‘dual-oscillator model’ (see text).

Given its strong association with diabetes, insulin secretion by pancreatic beta cells (Figure 1) has been the subject of intense study for almost a century [10]. The primary secretory pathway of glucose-stimulated insulin secretion is associated with complex patterns of electrical activity across the plasma membrane, which allow Ca^2+^ ions to enter the cell and trigger the secretory machinery. This electrical activity is coupled to cell metabolism, which acts as a glucose sensor by raising the intracellular ATP:ADP ratio, causing K_ATP_ channels to close, depolarising the membrane and driving it towards its threshold for action potential initiation [11]. Mathematical models provide an ideal framework to investigate the complex interaction between metabolic and electrical pathways in beta cells over the diverse timescales at which these processes occur.

The majority of mathematical models of beta cell behaviour are based on the Chay–Keizer model [12]. This model, which describes electrical activity and Ca^2+^ dynamics, has subsequently undergone a plethora of modifications, including those to incorporate glycolytic and mitochondrial components. The primary goal of these models is to elucidate the mechanisms giving rise to pulsatile insulin secretion with a mean period of ∼5 min observed in rodents, dogs, and humans 13, 14. To this end, many models consider oscillations in Ca^2+^ and metabolic activity under the assumption that one of these essentially sets the overall period of the pulses ([15] and references therein). However, the development and subsequent analysis of the dual-oscillator model [16] highlighted that these two mechanisms may actually work cooperatively to generate rhythmic insulin secretion (i.e., that Ca^2+^ and glycolytic activity can oscillate independently of one another but together give rise to oscillations on the timescale typically observed in experiments). This model has thus been an invaluable tool for studying the interactions of these processes and highlights the importance of understanding the timescales over which they occur 15, 17. The dual-oscillator model has since been modified to incorporate Ca^2+^ feedback to glycolytic activity. This improved integrated oscillator model 15, 18 further highlights that neither oscillations in Ca^2+^ nor metabolism establish the overall rhythmicity in beta cells by themselves 19, 20 and exemplifies how models can be developed in light of new experimental evidence.

One of the striking features of beta cells is that within islets they exhibit tight synchronisation of regular oscillations in electrical activity, while isolated cells oscillate irregularly ([21] and references therein). This phenomenon has been mathematically modelled by considering the islet as a network of beta cells. Under the heterogeneity hypothesis [22], variability in individual cells is ‘smoothed’ by intercellular interactions so that the network may be thought of as the average of the cells in it. This has led to the idea that islets are essentially a **syncytium**, with no single cell dictating the overall network response. However, this notion has been challenged by novel optogenetic experiments that show that silencing the activity of a single (specific) cell can disrupt electrical rhythms across the entire islet [23]. The presence of these so-called *hub* cells can be understood through the application of computational graph theory to the islet. Graph theoretic models place importance on the presence and nature of interactions within islets rather than the dynamics of individual beta cells [24]. Such models emphasise the dependency of these interactions on the extracellular concentrations of glucose [25] and that heterogeneous coupling could give rise to networks supporting hub cells [26], features that would be difficult to understand without an underlying model. Despite the success of using graph theory in this system, there is currently no experimental nor mathematical model that explains the results from the hub cell silencing experiment, but it is likely that combining the two approaches will be necessary to do so.

Alongside secretory deficiencies, **insulin resistance** is one of the primary mechanisms associated with the development of type 2 diabetes [27]. To investigate this, a recent phenomenological model [28] describes whole-body responses to insulin resistance including upregulation of beta cell function on short and medium timescales and changes to beta cell mass over longer timescales. Importantly, the model predicts the effect of temporary weight gain and loss as well as medical procedures such as gastric bypass surgery. The study introduces the notion of a threshold for decreases in insulin sensitivity: small decreases can be compensated for effectively whereas larger decreases cannot. In particular, the model highlights how feedback mechanisms to counter insulin resistance can contribute to the development of diabetes once the threshold has been crossed. The related concept of personal fat thresholds [29] is already being used to develop diet plans for diabetic patients; mathematical modelling has the potential to further support such interventions. Critically, analysis of the mechanisms in the model that establish the threshold explain why preventing diabetes is significantly easier than reversing it, exemplifying how models can be used not only to design therapeutic interventions (see Box 1 for an example), but also to direct public policy.

Box 1 **The Artificial Pancreas**

The ultimate aim of treatment in diabetes is to achieve glycaemic control; that is, to keep blood glucose concentrations within a certain band [30]. For individuals with type 1 diabetes, whose islets have an impaired ability to secrete insulin owing to the autoimmune destruction of their beta cells, exogenous insulin is typically administered preceding mealtimes in anticipation of spikes in blood glucose levels. Currently, the dose of insulin to be administered is predicted by estimation of the carbohydrate content of the proposed meal [31]. In addition, these individuals must monitor their glucose levels throughout the day using glucometers to prevent them from entering either hypo or hyperglycaemia. Developments in technology such as continuous glucose monitors and dose-adjusted insulin pumps offer the possibility of closed-loop control over blood glucose levels via their integration into an artificial pancreas 32, 33. Early tests of the artificial pancreas have been promising 34, 35, 36, 37 and the prospect of using mathematical models to understand the dynamics and feedback between glucose, insulin, glucagon, and other hormonal systems offers a powerful tool to support biomedical engineering advances. Importantly, mathematical models can expose inherent timescales in biological systems, the understanding of which is crucial for effective control. To this end, mathematical models of blood glucose–insulin dynamics can be used to design control that is predictive as well as reactive to blood glucose variations in post-prandial and fasting periods 38, 39, 40. The possibility of further development of control methods using Kalman filters opens avenues for the tailoring of parameters of the underlying models to the individual, with an ultimate aim of achieving a personalised treatment plan [41].

Alt-text: Box 1

## The Hypothalamic–Pituitary–Adrenal (HPA) Axis: A Choreography between Hormone Rhythms and the Stress Response

**Figure 2:**
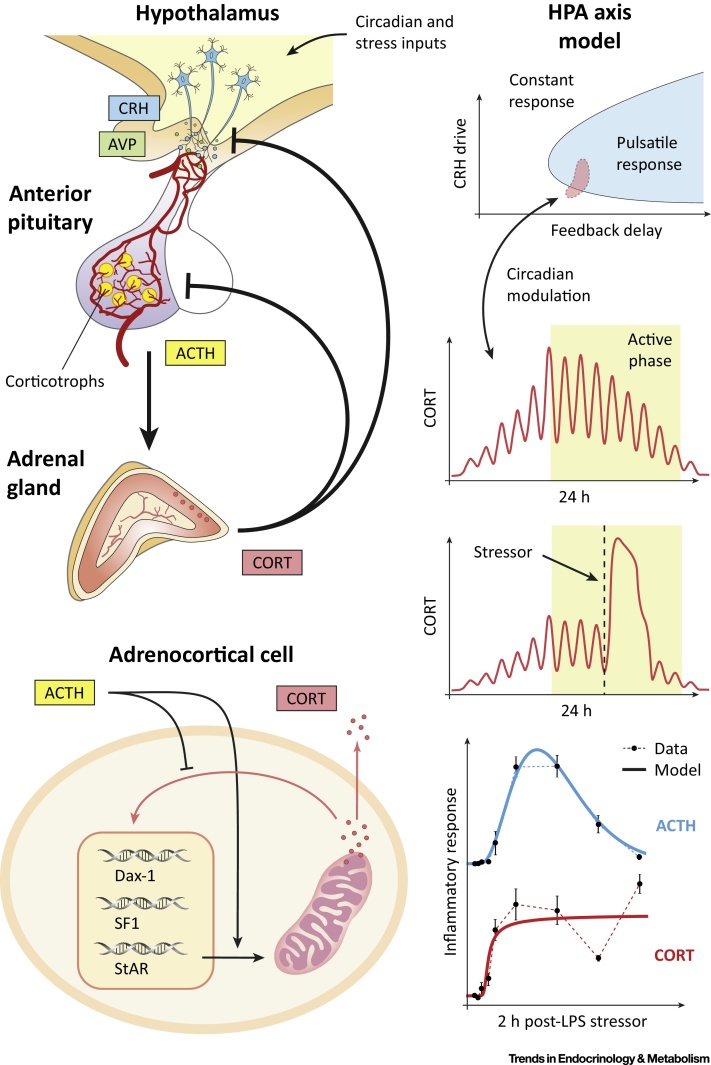
The Hypothalamic–Pituitary–Adrenal (HPA) Axis. Endogenous glucocorticoids (CORT) are vital hormones involved in many physiological processes that are key to homeostasis and survival (e.g., mediating the stress response, anti-inflammatory and immunosuppressive effects, regulation of glucose expenditure). The circulating levels of CORT are controlled by the HPA axis. Corticotropin-releasing hormone (CRH) and arginine vasopressin (AVP) stimulate the release of adrenocorticotropic hormone (ACTH) from the pituitary. ACTH in turn stimulates the adrenal glands to synthesise CORT, which further regulates its own synthesis through an intra-adrenal feedback loop. Within the HPA axis, CORT acts to inhibit ACTH in the pituitary as well as CRH and AVP in the hypothalamus, creating a dual negative-feedback loop. Combined mathematical and experimental studies have demonstrated that the tightly coordinated release of ACTH and CORT in ultradian pulses, observed under normal physiological conditions, is governed by this negative feedback [47]. These pulses have been shown to play an important role in the optimal responsiveness of glucocorticoid-sensitive neural processes. However, under pathological conditions (e.g., inflammation, chronic stress, neurological dysfunction) or ageing these pulsatile dynamics are altered and the tight synchrony between ACTH and CORT becomes significantly disrupted [54].

The body’s response to stress is mediated by several hormones, a crucial one being cortisol. Cortisol belongs to a group of glucocorticoid steroid hormones with a broad spectrum of context-dependent effects. Because they are rapidly secreted in response to physical and psychological stressors, they are commonly known as stress hormones. In the clinic, synthetic glucocorticoid hormones are widely prescribed for their anti-inflammatory effects as well as in hormone replacement therapy [42]. The circulating levels of glucocorticoids – cortisol in humans, corticosterone in rodents (CORT) – are dynamically controlled by the activity of the hypothalamic–pituitary–adrenal (HPA) axis (Figure 2), which is characterised by the rhythmic secretion of corticotropin-releasing hormone (CRH) and arginine vasopressin (AVP) from the paraventricular nucleus of the hypothalamus (PVN), adrenocorticotropic hormone (ACTH) from the pituitary, and CORT from the adrenal glands. Despite cumulative evidence showing the importance of CORT rhythms for immunological, cognitive, reproductive, and metabolic functions 42, 43, little attention has been paid to developing the dynamic aspects of glucocorticoid drug therapies. From a theoretical point of view, understanding how the HPA axis sustains rhythmic activity while simultaneously eliciting fast, transient, and proportionate responses to stressors constitutes a major challenge.

One of the key steps in understanding the dynamic activity of the HPA axis relates to the causal relationship between ACTH and CORT secretion. A pioneering mathematical model addressed this challenge by accounting for several steps of the signalling pathway: the activation of a putative ACTH receptor in the membrane of adrenocortical steroidogenic cells, its relay via cAMP in the cytosol, the mitochondrial import of cholesterol (the substrate for CORT biosynthesis), and the synthesis and secretion of CORT [44]. The model was fitted to adrenal secretory rates of cortisol and blood ACTH concentrations measured in dogs subjected to intravenous infusions of ACTH. Importantly, this model predicted changes in adrenal sensitivity between small versus large pulses of ACTH, a phenomenon that has been further identified and investigated in other mammals. Subsequent models considered the feedback loops that glucocorticoids exert at the level of the pituitary and hypothalamus 45, 46. These models offered qualitative predictions of feedback-generated ultradian oscillations in CORT levels and suggested possible ways to include circadian modulation. In this sense, [45] showed that coupling this feedback mechanism with a central nervous system-driven pulse generator enables both ultradian and circadian variability in hormone secretion. These early models also aimed at explaining specific physiopathological changes such as stress, infusion of synthetic glucocorticoids, and adrenalectomy. Interestingly, the model in [46] also proposed a bistability mechanism that would explain the allostatic transition of the HPA axis subjected to chronic stress.

Although the models in 45, 46 demonstrate the possibility of ultradian oscillations generated through negative feedback, the predicted frequency of these oscillations significantly differs from the near-hourly oscillations observed in humans. It was not until the work by Walker *et al*. [47] that the mechanisms underlying ultradian oscillations were correctly predicted as originating from the negative feedback loops between the pituitary and adrenal glands, while the hypothalamic drive provides the source of circadian modulation. This model predicted near-hourly oscillations of ACTH and CORT secretion supported by *in vivo* data, even in the presence of a constant hypothalamic CRH signal. Subsequent experiments confirmed this model prediction 48, 49, which demonstrated that a previously hypothesised hypothalamic ‘pulse generator’ [50] is not essential to generate ultradian glucocorticoid oscillations.

While recent mathematical models of the HPA axis have focused on the role of glucocorticoid dynamics in mental health 51, 52, others have investigated the stress response, the role of nuclear receptors, and inflammation 53, 54, 55. Understanding how healthy adrenal glands achieve rapid CORT secretion while simultaneously preventing their uncontrolled release in response to stressors is key to explaining the dysregulation observed in endocrine disorders such as Addison’s disease and Cushing’s syndrome (Box 2). In this direction, the work in [55] combined experimental physiology and mathematical modelling to predict how surges of ACTH may be decoded by the adrenal gland, hypothesising that the control mechanism may comprise an intra-adrenal negative feedback loop mediated by the glucocorticoid receptor. The organisation of the molecular mechanisms involved in such intra-adrenal regulation was postulated in [54], distinguishing between slow genomic and fast non-genomic signalling pathways. These mechanisms were mathematically modelled as a regulatory network that not only predicted the transient dynamic responses observed during the stress response but explained how the adrenal glands can decode ACTH pulses of different magnitudes, including those observed during inflammation.

Box 2 **The Chronobiology of Stress in Health and Disease**

An urgent need for mathematical modelling is emerging in the early diagnosis and treatment of steroid-related disorders. The adrenal glands produce hormones that have important roles in the regulation of inflammation, metabolism, blood pressure, fertility, and mental health. Levels of hormones normally fluctuate during the day and respond rapidly to stressors (both physical and psychological). In all healthy individuals, fluctuations are organised rhythmically [8]. However, patients with certain endocrine conditions (e.g., Cushing’s, Addison’s, primary aldosteronism) experience disruptions of this rhythmicity that deviate from the normal variability in healthy subjects. This is important since diagnosis of these conditions is difficult with current clinical tools, which rely on single-time-point sampling from blood. Consequently, diagnosis is often delayed, and this may result in inadequate or inappropriate treatment, which results in further deterioration of the patient’s health and increased costs. By accounting for the intrinsic dynamic hourly-to-daily characteristic of hormone rhythms, mathematical methods can achieve fast classification of pathological hormone profiles versus normal physiological variability with quantified uncertainty. Furthermore, mechanistic modelling of the active dynamic regulation of the stress response [55] can help in understanding the body’s expected demand for cortisol in response to different ranges of stress during health and disease. This is particularly important for patients requiring lifelong steroid replacement therapy, and recent research has demonstrated that patients using a novel infusion method have their dynamic cortisol levels restored to normal 56, 57. However, it remains a challenge to design an active control of hormone infusion that dynamically responds to everyday stressors. In this direction, computational algorithms developed from mathematical models can assist the development of dynamic drug delivery devices. Similar modelling approaches may have a natural application in chronotherapies such as timing the treatment of chronic inflammatory diseases, stress-related fertility interventions, and the management of metabolic conditions.

Alt-text: Box 2

## The Reproductive Axis: Uncovering the Mechanisms of GnRH Pulsatility

**Figure 3:**
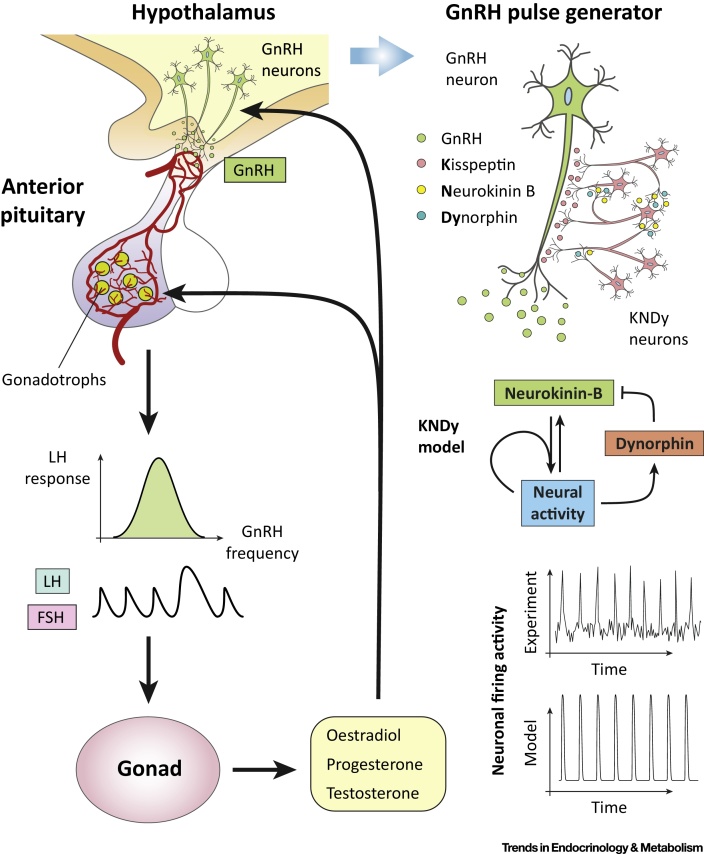
The Hypothalamic–Pituitary–Gonadal (HPG) Axis. Reproduction is controlled by the HPG axis. Gonadotropin-releasing hormone (GnRH), secreted by GnRH neurons located at the hypothalamus, stimulate the release of gonadotropin hormones [luteinizing hormone (LH) and follicle-stimulating hormone (FSH)] from the pituitary. The release of gonadotropins critically depends on GnRH pulsatile dynamics that are driven by hypothalamic neuronal networks. Gonadotropins act on the gonads, initiating processes involved in gametogenesis and ovulation and triggering the release of sex steroids (oestradiol, testosterone, progesterone) that feedback on the brain and pituitary gland to modulate GnRH and LH/FSH secretion dynamics. Mathematical models [64] have offered insight into how hypothalamic neurons coexpressing kisspeptin, neurokinin-B, and dynorphin control the pulsatile dynamics of GnRH secretion and how these pulsatile signals are decoded by single cells at the pituitary gland.

Hormone signals within the hypothalamic–pituitary–gonadal (HPG) axis (Figure 3) are critical for reproduction, with a key regulatory process being the pulsatile release of gonadotropin-releasing hormone (GnRH) from the hypothalamus onto the pituitary gland. Mathematical models have provided insight into how GnRH pulsatility controls the synthesis and secretion of gonadotropic hormones [luteinizing hormone (LH) and follicle stimulating hormone (FSH)] from the pituitary. Early experimental work on primates revealed the dependence of gonadotropin secretion on GnRH frequency by showing that pulsatile but not constant delivery of exogenous GnRH can restore gonadotropin secretion in animals with hypothalamic lesions [58]. It is now clear that gonadotropin secretion is suppressed when the GnRH frequency is either too high or too low, and this effect is mediated through complex signalling networks that allow cells to regulate the synthesis of LH and FSH differentially in response to GnRH frequency 59, 60. Several mathematical models related to GnRH signalling have been proposed [61] and a mechanistic model of the pathway has shown that the nonlinear relationship between gonadotropin secretion and GnRH pulse frequency is most likely due to the convergent feed-forward architecture of the network 61, 62. The model suggests that frequency decoding is primarily achieved due to the synergistic effect of multiple signalling pathways [e.g., the extracellular signal regulated kinase (ERK) pathway and the nuclear factor of activated T cells (NFAT) pathway] on the expression of gonadotropin-related genes. This contrasts with upstream negative feedback interactions (e.g., due to agonist-induced receptor internalization) that were previously thought to play a crucial role in frequency decoding. Instead, the model shows that feedback plays a different role, allowing the pituitary system to cope with cell–cell heterogeneity and process GnRH information more reliably [63].

At the level of the hypothalamus, a coarse-grained neuronal population model has advanced our understanding of how GnRH pulsatility is sustained and regulated [64]. The model draws on recent experimental work, which demonstrates the pivotal role of neuropeptide signalling within the arcuate nucleus kisspeptin population for GnRH pulse generation 65, 66. The model supports the idea that the kisspeptin population drives GnRH pulses by postulating that it operates as a **relaxation oscillator** due to neuropeptidergic negative and positive feedback interactions mediated by neurokinin B and dynorphin, respectively. Furthermore, the model predicts that pulsatile dynamics depend on basal activity levels in the kisspeptin population and highlights the tipping-point behaviour of the system as basal activity increases. Using optogenetics, these model predictions were confirmed *in vivo*, showing that pulses can be directly controlled in **oestrous** mice by selectively exciting kisspeptin neurons in the arcuate nucleus with continuous low-frequency (1 Hz and 5 Hz) light stimulation [64]. Thus, this is yet another example of how even simple phenomenological models can lead to useful and experimentally testable insights.

Mathematical modelling has also been employed to understand the macroscopic processes involved in follicular development [67]. Although gonadotropins are known to control the development of ovarian follicles and their secretory activity, little attention has been given to sex steroid secretion and how it feeds back to upstream components of the HPG axis modulating GnRH and gonadotropin secretion. These feedback interactions underpin the ovarian cycle and have a critical role in women’s physiology and reproductive health, thus representing a unique opportunity for experimental physiologists, clinicians, and mathematical modellers alike [68].

## Hybrid Systems: A New Paradigm to Establish How the Parts Contribute to the Whole

Like alpha and beta cells in the pancreas, the five endocrine cell types of the anterior pituitary generate electrical activity in the form of spikes and bursts [69]. Electrical activity brings Ca^2+^ into the cells through ion channels, which triggers hormone secretion and stimulates vesicle refilling. In the absence of hypothalamic signals, pituitary gonadotrophs fire sharp spikes at a slow rate, releasing very little hormone. By contrast, lactotrophs and somatotrophs fire in bursts, which are longer electrical events than spikes. Bursts provide more time for Ca^2+^ to enter cells, so lactotrophs and somatotrophs have a high basal rate of hormone release [70]. While pituitary cells have similar amounts of most voltage- and Ca^2+^-activated channels, they differ in the amount of large-conductance potassium (BK) channels. Lactotrophs and somatotrophs have a high density of BK channels, while gonadotrophs have very few [71]. This is paradoxical because BK channels are repolarising channels (in neurons and other cell types). BK channels typically open quickly during an action potential, reducing its duration. However, in pituitary cells like somatotrophs and lactotrophs, BK channels seem to increase event duration, turning spikes into bursts. This prompts the question of whether gonadotrophs would burst if they expressed BK channels.

**Figure 4:**
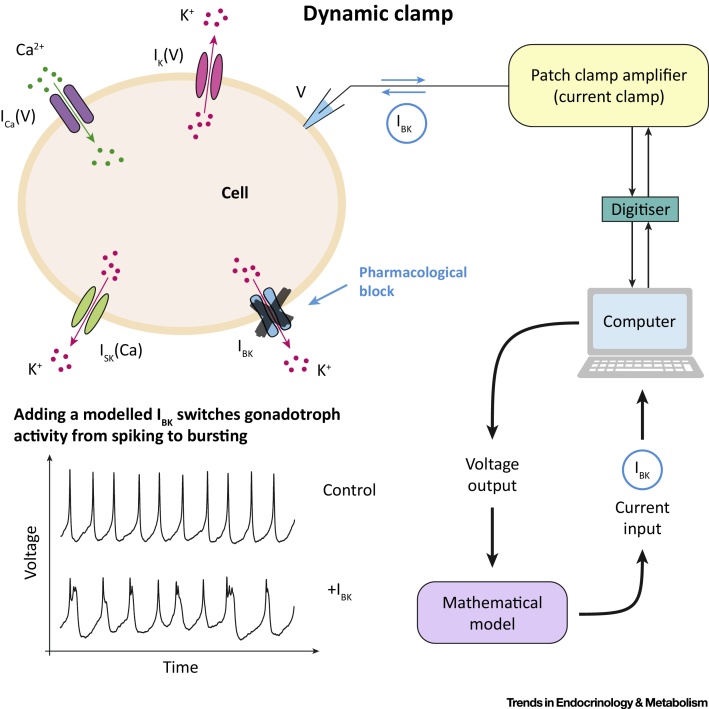
The Dynamic Clamp: A Real Time, Simultaneous Modelling and Experimental Hybrid System. Traditionally, mathematical models have been integrated with experiments via an iterative process: predictions from models are tested against results from appropriate experiments and the models are then updated to address any discrepancies between the two. While this has been, and continues to be, a fruitful endeavour in many cases, hybrid experiments allow the two to be brought together in a real time and interactive fashion. Hybrid systems enable us to manipulate the values of key parameters with the freedom of a mathematical model. At the same time, the effects of these manipulations are observed in real biological systems. One example of a hybrid system is the dynamic clamp protocol for electrically excitable cells [87]. In this system, a mathematical model is used to provide a command signal to the cell from which an electrical recording is being taken. Importantly, since the real-time membrane potential of the cell can be provided to the model, this can be used to inject signals that mimic ionic currents that may or may not be present in the real cell. In this way, parameters associated with these currents can be manipulated, or entirely different channels can be incorporated into the cell. Recently, this method has been used to determine the role of large-conductance potassium (BK) channels in shaping the electrical activity of pituitary cells (see text) [73].

A mathematical model predicted that assimilation of BK channels into gonadotroph electrical activity can switch its firing dynamics from spiking to bursting. By opening quickly at the beginning of an action potential, BK channels limit the activation of other, slower K^+^ channels, which in turn prevents these channels from repolarising the cell [72]. Analysis of the model suggests that this effect is robust to changes in the expression of other ion channels [73] but leaves open the question of whether fast BK current activation promotes bursting in real cells. This problem was elegantly solved through the dynamic clamp technique (Figure 4). Bursting lactosomatotroph cells first had their BK channels blocked by a channel antagonist, resulting in a switch from bursting to spiking in most cells. Then, a BK current calculated in real time from a mathematical model was added back to the cells via a computer-assisted dynamic clamp. This made the cells switch back to bursting and, importantly, it occurred only if the modelled BK current was fast enough, demonstrating that the mechanism identified by the mathematical model was correct. Finally, use of the dynamic clamp to add a model BK current into spiking gonadotrophs made these cells switch to bursting, demonstrating that the difference between electrical activity patterns in lactosomatotroph and gonadotroph cells could be explained by the presence or absence of BK channels [73].

The dynamic clamp was instrumental in establishing the role of BK channels, by linking a model-based mathematical mechanism to real pituitary cells. It illustrates the power of hybrid systems to combine experiments and modelling. Another elegant example of such a system was developed by Dhumpa *et al*. [74] to show that islets of Langerhans can synchronise their insulin secretion through feedback from the liver. To do so, they introduced islets loaded with a fluorescent Ca^2+^ indicator into a microfluidic chamber and interfaced the global Ca^2+^ signal from the islet population with a mathematical model of glucose release by the liver in response to insulin. The modelled glucose level was then delivered back to the islet chamber. Without liver feedback, the islets produced independent oscillations. As soon as the feedback was turned on, however, the islets began to synchronise, as evidenced by the resulting global Ca^2+^ oscillation, out of phase with the resulting glucose oscillation. This demonstrated that the liver might act as a coordinator of activity in the islet population and enabled testing of the effectiveness of this coordination as the speed of the liver feedback was varied. Thus, hybrid systems allow us to determine the role played by components of a biological system, by controlling key parameters of such components, particularly the timescale on which they operate.

## Concluding Remarks and Future Perspectives

The complexity of endocrine systems is evidenced by the number of molecular interactions occurring at multiple levels of organisation that are necessary to achieve robust control of hormone secretion. Strikingly, many endocrine axes exhibit the same control strategies to regulate hormone levels within a homeostatic range: feedback loops, network organisation of components, and collective behaviour that cannot be explained solely by investigating the dynamics of individual cells. Here, we have reviewed recent examples from three major endocrine axes where mathematical models have delivered insight about dynamic behaviour that was difficult to interpret solely by looking at the experimental data. The relevance and timeliness of using mathematical tools to understand these control strategies is largely driven by the urgency of understanding their dysregulation in reproductive, metabolic, and stress-related conditions, including complex psycho-immunoneuroendocrine disorders 6, 75, 76, 77.

The key role of hormone dynamics in health and disease has suggested future research avenues at the interface of mathematical modelling and experimental neuroendocrinology. In this sense, frequency encoding and decoding mechanisms underlying pulsatile hormone secretion remain an understudied area [77]. For instance, ultradian hormone stimulation is known to induce glucocorticoid receptor-mediated pulses of gene transcription [78], and there exists a growing realisation that understanding how the dynamics of glucocorticoid signalling affects gene regulation is key to the design of effective chronotherapies 79, 80. The development of such understanding will be likely to involve modelling the role of hormone pulsatility on continuous dynamic equilibration and stochastic dynamic interactions at the level of DNA binding [75].

Another burgeoning area of research is the crosstalk interactions between endocrine axes. For instance, hypercortisolism induced by chronic stress, Cushing’s syndrome, or medication is a known risk factor for the development of diabetes. This has prompted investigations on the links between glucocorticoid dynamics and insulin secretion and resistance 81, 82, 83. Similarly, a mathematical model linking the HPA and metabolic axes describes a way in which circadian glucocorticoid oscillations regulate a transcriptional circuit underlying adipocyte differentiation [84], suggesting mechanisms by which conditions that disrupt pulsatile glucocorticoid secretion could lead to obesity. By contrast, insulin-induced hypoglycaemia is an acute stressor that both significantly activates the HPA axis and inhibits pulsatile LH secretion in rats [85], evidencing crosstalk interactions between the metabolic, stress, and reproductive axes. Gender differences in endocrine regulation are also being investigated via mathematical methods, as suggested by a model exploring the effects of testosterone on the HPA axis response to stress [86].

While in most experimental research it is sufficient to ‘let data speak for itself’, existing experimental protocols as applied to complex endocrine phenomena often struggle to combine data at different levels of organisation. As a result, the mutual interactions between factors underlying endocrine regulation and the different timescales at which they occur are often ignored. This is where mathematical models offer a solution to interpret the data and gain insight on the underlying dynamics. Moreover, models help us think beyond the limits of ‘what we can do’ at the laboratory bench and start asking ‘what if’ questions. This not only stimulates creative interdisciplinary collaborations but also advances the field by replacing a static, snapshot view of endocrine function with one where complex, multiscale regulation underpins hormone dynamics (see Outstanding Questions).

Outstanding Questions Hormone pulsatility in endocrine systems is ubiquitous, but whether this is an optimal solution compared with constitutive secretion needs to be investigated. Is there an energetic advantage in the reduced amount of hormone needed for pulsatile signalling?

Compared with constant hormone levels, rhythmic hormone secretion contains more information in the form of, for example, amplitude and frequency. How is this information encoded and how do different tissues read the same blood signalling message in different ways?

Hormone rhythms are known to affect gene expression at different timescales and in a context-dependent way. At a cellular level, how are chromatin responses determined by the pattern of nuclear receptor activation?

How does chronodisruption, especially between different local and central pacemakers, cause disease?

Can our increased knowledge of hormone dynamics be used in therapeutics that improve patient care?

## Glossary

**Allostasis**: the adaptive processes by which a physiological regulatory system re-establishes homeostasis (typically with increased fragility) to compensate for physiological disruptions.
**Bistability**: the coexistence of two stable equilibrium states that, under certain conditions, may be observed in a dynamical system.
**Chronomedicine**: a novel approach to medicine that focuses on understanding the natural rhythms of the body for the prevention, diagnosis, and treatment of diseases. Associated terms are chronodisruption (when the timing of perturbations is key to the disruption of a physiological rhythm), chronotherapy (when the timing of treatment is key to restoring health), and chronobiology (when referring to rhythms not exclusive to humans).
**Circadian**: a biological rhythm displaying an oscillation period of about 24 hours.
**Insulin resistance**: a decrease in the sensitivity of target tissue to insulin.
**Nonlinear response**: the response of a regulatory system in which the change of the output is not proportional to the change in the input.
**Oestrous**: refers to the onset of a reproductive cycle in most mammals.
**Relaxation oscillator**: an oscillator that achieves its rhythmicity from repetitive cycles of accumulation and discharge (e.g., electrical activity, neurotransmitter concentration).
**Robustness**: the ability of a system to preserve its dynamic behaviour while coping with perturbations.
**Sensitivity**: the ability of a system to respond rapidly (and/or with large excursions) to stimuli.
**Syncytium**: the conceptualization of a cohort of multiple cells exhibiting such a degree of interconnectedness and synchronised behaviour that they may be understood as if they were a single cell (e.g., multinucleate cell).
**Ultradian**: a biological rhythm displaying an oscillation period of less than 24 hours..
